# Variability of Chemical Compositions and Antimicrobial and Antioxidant Activities of* Ruta chalepensis* Leaf Essential Oils from Three Palestinian Regions

**DOI:** 10.1155/2017/2672689

**Published:** 2017-11-05

**Authors:** Nidal Jaradat, Lina Adwan, Shadi K'aibni, Abdel Naser Zaid, Munqez J. Y. Shtaya, Naser Shraim, Mohyeddin Assali

**Affiliations:** ^1^Department of Pharmacy, Faculty of Medicine and Health Sciences, An-Najah National University, Nablus, P.O. Box 7, Nablus, State of Palestine; ^2^College of Pharmacy, Nursing and Health Professions, Birzeit University, P.O. Box 14, Birzeit, State of Palestine; ^3^Center of Birzeit University Testing Laboratories, Birzeit University, P.O. Box 14, Birzeit, State of Palestine; ^4^Faculty of Agriculture and Veterinary Medicine, An-Najah National University, P.O. Box 7, Nablus, State of Palestine

## Abstract

**Introduction:**

Interest in essential oils was recently revived with their popularity increasing in medicine, pharmacy, and aromatherapy. This study was performed to identify the chemical compositions of the essential oil of* Ruta chalepensis* growing wildly in three regions in Palestine and to assess and compare their antimicrobial and antioxidant activities.

**Methods:**

Identification of the essential oil was performed by gas chromatography coupled with mass spectrometry (GC-MS). Antimicrobial activity was tested against* Staphylococcus aureus*,* Escherichia coli*,* Pseudomonas aeruginosa*, Methicillin-Resistant* Staphylococcus aureus*, and* Candida albicans* by using minimum inhibitory concentration (MIC) assay, while antioxidant activity was analyzed by using the 2,2-diphenyl-1-picrylhydrazyl (DPPH) free radical scavenging method.

**Results:**

The essential oils of* R. chalepensis* from Jerusalem and Hebron regions have almost identical components; the major compounds identified were linalyl acetate and *β*-linalool; these essential oils exerted potential antioxidant and antibacterial activities. On the other hand, the major components of the plant essential oil from Jenin region were 2-undecanone and 2-nonanone, which exhibited potential antifungal activity.

**Conclusions:**

The phytoconstituents and antioxidant and antimicrobial properties of the essential oil of* R. chalepensis* from different regions in Palestine were established in this study. The obtained results indicate possible applications for* R. chalepensis* in the treatment of various infectious and noninfectious diseases.

## 1. Introduction

In the last three decades, a huge progress in medicinal plants research has been observed. In fact, the global interest towards the use of herbal remedies has created a huge need for information about the uses and the therapeutic properties of these plants [1]. Essential oils are among the most interesting components of herbals and plants. These essential oils are rich in hydrocarbon compounds that have oxygenated, hydrogenated, and dehydrogenated functional groups. Most of these chemicals are monoterpenoid or sesquiterpenoids. They are odorous principles, which are found in various plants parts and evaporate at ordinary temperature [2, 3]. They are used as therapeutic agents in ethno, conventional, and complementary alternative medicines particularly as analgesics, anti-inflammatory, antispasmodic, local anesthetic, anthelmintic, antipruritic, and antiseptic as well as many other therapeutic uses [4–7].

Nowadays, essential oils are used broadly in medicine and cosmeceutical and pharmaceutical industries and as flavoring agents and preservatives in food industry and design [8, 9]. In addition, it is scientifically accepted that natural antioxidants exert health promoting effects and their consumption as food or as food additives or as nutraceuticals and supplements have been greatly promoted worldwide. A complex mixture of antioxidants may account for improvement of cardiovascular health and decreased incidence of cancer in individuals who consume more of these substances [10–12]. In elderly subjects, a higher intake of antioxidants is linked with improved health compared to subjects consuming less fruits and vegetables in their diet [13–15].

On the other hand, many food industries utilize synthetic chemical preservatives to extend the shelf life of the produced commercial food products. Unfortunately, the chronic intake of these chemicals may cause carcinogenic or toxic effects to consumers. Accordingly, a growing interest in the discovery of new natural antimicrobial and antioxidant agent has been observed in order to avoid or minimize the undesirable consequences and side effects related to the consumption of chemical preservatives [16–18]. In fact, the so-called hurdle technology, combining antioxidant and antimicrobial agents, has gained wide acceptance within the food and pharmaceutical industries [19].

Combining two or more compounds could be more effective for improving antioxidant and antimicrobial activity and could offer a synergistic potential. However, the use of phytochemicals to prevent foodborne microbes is poorly studied [20–22]. Many scientific investigations concluded that the chemical compositions and yields of essential oils may be affected by environmental factors such as seasonal and climate variations, geographical conditions, and growth stages of the plant in addition to the genetic factors for the same plant species [23–30].

*Ruta chalepensis* L. (Rutaceae), commonly known as Rue or Fringed rue, is a wild perennial herbaceous shrubs which is widely distributed in the Mediterranean Sea regions. It usually grows on the rocky slopes of mountains. It is has glabrous, alternate bipinnatisect leaves with narrow oblong lanceolate or obovate segments and cymose inflorescence [31]. It is the only rue species mentioned and covered in the Flora Palaestina and even mentioned in the Bible under the Greek name Pigam, closely cognate with the Arabic name Figam which is used as flavor for honey, wine, and olive oil [32]. Due to its high content of amino acids, saponins, phenols, flavonoids, alkaloids, and furocoumarins in the leaves and young stems,* R. chalepensis* has pleiotropic pharmacological activities [33]. It has been used widely in various folk medicines as analgesic and antipyretic and for treatment of mental disorders, convulsions, rheumatism, dropsy, neuralgia and bleeding disorders [34–38].

In the Palestinian traditional medicine, it has been used for treatment of asthma, renal colic, arthritis, rheumatism, backache, skin bacterial and fungal diseases, eye inflammation and ear infection. Besides that, it has been used as antitussive, antispasmodic, anti-inflammatory, antilice, sedative, and bronchodilator and also for treatment of snake bites [39–41].* R. chalepensis* essential oil showed potential repellent activity against* Aegorhinus superciliosus* and* Aedes albopictus* [42]. Therefore, the aims of the current study were to identify the chemical composition of* R. chalepensis* growing wildly in three regions of the West Bank area of Palestine. Afterwards, screening and comparison of their antimicrobial and antioxidant activity were conducted.

## 2. Materials and Methods

### 2.1. Chemical Reagents

Trolox (6 hydroxy-2,5,7,8-tetramethylchroman-2-carboxylic acid) was purchased from Sigma-Aldrich, Denmark, 2,2-diphenyl-1-picrylhydrazyl (DPPH) was obtained from Sigma-Aldrich, Germany, and methanol was from Loba Chemie, India, and they were used to evaluate the antioxidant activity of* R. chalepensis* essential oil.

Nutrient broth was purchased from Himedia, India, and dimethyl sulfoxide (DMSO) was purchased from Riedel-de Haen, Germany, which were used for the screening of the antimicrobial activity of* the R. chalepensis* essential oils.

### 2.2. Instrumentation

Gas chromatography mass spectrometry (GC-MS) (QP-5000 GC-MS Shimadzu, Japan) was used to assess the chemical composition of the essential oils. Ultrasonic-microwave cooperative extractor/reactor (CW-2000, China) was used to extract the essential oils. UV-visible spectrophotometer (Jenway 7315, England) was used to assess the antioxidant activity. Grinder (Moulinex model, Uno, China) was used to mill the dried plants. Balance (Radw ag, AS 220/c/2, Poland) was used for sensitive weighing of the samples. Filter papers (Macherey-Nagel, MN 617 and Whatman no. 1, USA), micropipettes (Finnpipette, Finland), incubator (Nuve, Turkey), syringe filter 0.45 *μ*m pore size (Microlab, China), and 96-well plates (Greiner bio-one, North America) were used for antimicrobial tests.

### 2.3. Collection and Preparing Plant Materials

The leaves of* R. chalepensis* were collected during its flowering time, in April 2015, from three regions of Palestine; Jerusalem (the middle part of the West Bank), Hebron (the southern part of the West Bank), and Jenin (the northern part of the West Bank). The plant taxonomical classification was carried out by Pharmacognosist Dr. Nidal Jaradat. Voucher specimens were deposited in the Pharmacognosy Laboratory, Faculty of Medicine and Health Sciences, An-Najah National University, under the code number: Pharm-PCT-2084.

To extract the essential oil, the methods in previously published studies were followed; the leaves of* R. chalepensis* were separated carefully and then washed twice with distilled water. The washed leaves were dried for 10–14 days in the shade at room temperature to avoid damage and to minimize cross-contamination of the separated leaves. Finally, the dried leaves were grounded well and the powder obtained was stored in cloth bags for future use [43].

### 2.4. Essential Oils Extraction

The essential oils of the three samples of* R. chalepensis* plant were isolated by using microwave-ultrasonic method which was described by Jaradat with minor modifications [44]. The powder suspension was exposed to ultrasonic waves to improve the isolation process. In this study, the apparatus consisting of a microwave oven combined with an ultrasonic extractor was used. About 100 g of the dried leaves powder was suspended in about 500 ml deionized water into a 1 L round bottom flask and placed in this apparatus and connected with Clevenger apparatus. The power of the microwave-ultrasonic extractor apparatus was adjusted at 1000 W. The ultrasonic power of the apparatus was adjusted at its maximum power as well (50 W with a frequency of 40 kHz). The isolation process using this apparatus was conducted for 10 min at 100°C. This process was repeated for three times for each plant sample. The obtained essential oil was collected into a clean beaker, chemically dried, and stored in the refrigerator at 2–8°C [7]. The obtained* R. chalepensis* essential oils yield was 0.6% v/w from the Jenin sample, 1.3% v/w from the Hebron sample, and 1.6% v/w from the Jerusalem sample.

### 2.5. GC-MS Analysis

The GC-MS chromatograms were recorded using Shimadzu QP-5000 GC-MS. The GC was equipped with Rtx-5 ms column (30 m long, 0.25 *μ*m thickness, and 0.25 mm inner diameter). Helium was used as a carrier gas at a flow rate of 1 ml/min. The injector temperature was 220°C. The oven temperature was programmed from 50°C (1 min hold) at 5°C/min to 130°C and then at 10°C/min to 250°C and was kept isothermally for 15 min. Transfer line temperature was 290°C.

For GC-MS detection, an electron ionization system, with detector volts of 1.7 KV, was used. A scan rate of 0.5 s and scan speed 1000 amu/sec were applied, covering a mass range 38–450 M/Z.

### 2.6. Identification of Components

The chemical constituents of the essential oils were recognized by comparing their MS with the reference spectra in the mass spectrometry data center of the National Institute of Standards and Technology (NIST) and by comparing their retention indices and Kovats indices in the literature. The quantitative data were obtained electronically from area percentages and integrated peaks without the use of correction factor [45].

### 2.7. Antioxidant Activity

Stock solutions at a concentration of 1 mg/ml in methanol and Trolox were prepared from* R. chalepensis* essential oils that were collected from three Palestinian regions. Each one of these stock solutions was diluted in methanol to prepare 12 of the working solutions with the following concentrations: 1, 2, 3, 5, 7, 10, 20, 30, 40, 50, 80, and 100 *μ*g/ml. A freshly prepared DPPH solution (0.002% w/v) was mixed with both methanol and with each of the above-mentioned working solutions at 1 : 1 : 1 ratio. In addition, a negative control solution was prepared by mixing the mentioned DPPH solution with methanol in 1 : 1 ratio. All of these solutions were incubated at room temperature in a dark cabinet for 30 min. By the end of the incubation period, the optical density of these solutions was determined spectrophotometrically at a wave length of 517 nm using methanol as the blank solution.

The antioxidant activity of Trolox and* R. chalepensis* essential oils was estimated by using the following formula:

(1) $$\begin{array}{c} \%\;\;Inhibition\;\;of\;\;DPPH\;\;activity=\frac{A-B}{A}\times 100\%, \end{array}$$

where *A* and *B* represent the absorbance of the blank and the oil, respectively.

The antioxidant half-maximal inhibitory concentration (IC_50_) for each of the studied* R. chalepensis* essential oils and Trolox and their standard deviations were calculated by using BioData Fit edition 1.02 (data fit for biologist).

The antioxidant activities of* R. chalepensis* essential oils at the different concentrations mentioned above were expressed in terms of the antioxidant activity of the Trolox standard. This was determined by using the following equation:

(2) %  Inhibition  according  to  Trolox=Trolox  IC50volatile  oil  IC50×100%.

### 2.8. Antimicrobial Evaluation

The essential oils of* R. chalepensis* obtained from the three regions in this study were investigated for their antimicrobial activity. The antibacterial activities of* R. chalepensis* essential oils were examined against the growth of three references bacterial strains obtained from the American Type Culture Collection (ATCC) including* Staphylococcus aureus* (ATCC 25923),* Escherichia coli* (ATCC 25922), and* Pseudomonas aeruginosa* (ATCC 27853) as well as against the growth of a diagnostically confirmed clinical isolates Methicillin-Resistant* Staphylococcus aureus* (MRSA). The antifungal activity of* R. chalepensis* essential oils was examined against the growth of a diagnostically confirmed clinical isolates* Candida albicans*.

The antimicrobial activity of* R. chalepensis* essential oils obtained from the three regions used in this study was determined by using broth microdilution method as described previously [46].

Each one of the isolated* R. chalepensis* essential oils was dissolved in DMSO (5%) at a concentration of 132 mg/ml. The prepared* R. chalepensis* essential oils solutions were filter-sterilized and then were serially microdiluted (2-fold) eleven times in sterile nutrient broth. In 96-well plates, the dilution processes were carried out under aseptic conditions. In the microwells that were assigned to evaluate the antibacterial activities of the extracted* R. chalepensis* essential oils, the concentration of these oils ranged from 0.129 to 66 mg/ml. On the other hand, the concentrations of these essential oils in the microwells assigned to evaluate their antifungal activities ranged from 0.065 to 55 mg/ml. In these plates, microwell number 11 contained essential oil free nutrient broth, which was used as a positive control for microbial growth. In addition, the microwell number 12 contained essential oil free nutrient broth that was left uninoculated with any of the test microbes. This well was used as a negative control for microbial growth. Microwells number 1 to 11 were inoculated aseptically with the test microbes. At the time of inoculation, the final concentrations of microbial cells were about 5 × 10^5^ and 0.5–2.5 × 10^3^ colony-forming unit (CFU)/ml for the tested bacterial pathogens and* Candida albicans*, respectively. Each of the included microbes in this study was examined in duplicate for being inhibited by the* R. chalepensis* essential oils.

At 35°C all the inoculated plates were incubated and the incubation period lasted for about 18 hours for the plates inoculated with the test bacterial strains and for about 48 hours for the plates inoculated with* Candida albicans*. The lowest concentration of* R. chalepensis* essential oil, at which no visible microbial growth in that microwell was observed, was considered as the minimum inhibitory concentration (MIC) of the examined* R. chalepensis* essential oil. The MIC of gentamycin and amphotericin B were also determined in parallel experiments as positive controls for antimicrobial activity and all the established tests were performed in triplicate.

### 2.9. Statistical Analysis

The antioxidant IC_50_ values were determined in triplicate for the essential oils of* R. chalepensis* obtained from three different Palestinian regions. The results were expressed as means ± standard deviation (SD) and the obtained data were compared using ANOVA with multiple comparisons. All the data were considered statistically significant when the *p* value was <0.05. The statistical significance was represented as *∗* with *p* value < 0.05, *∗∗* with *p* value ≤ 0.001, and ∗*∗∗* with *p* value ≤ 0.0001.

## 3. Results

The phytochemical profile of the* R. chalepensis* essential oil components from Hebron region was almost similar to Jerusalem region and the total components of the essential oil were 96.75% and 96.3%, respectively. In addition, they contained major phytochemical classes such as (i) alcohol which represented 32.36% and 38.24%, respectively, (ii) ketone group 31.16% and 18.27%, respectively; and (iii) ester group which represented 29.51% and 35.05%, respectively. Meanwhile the essential oil from the same species growing in Jenin region had almost different components: the total essential oil represented 92.58% and the major represented class of phytoconstituents was ketonic products with 89.81% of total identified essential oil components. Moreover, 14 components were identified from* R. chalepensis* essential oil which was harvested from the mountains of Hebron. Regarding* R. chalepensis* essential oil which was obtained from the mountains of Jerusalem, 17 components were identified. Surprisingly, only 7 components were identified in the essential oil that was extracted from* R. chalepensis* of Jenin region and two phytochemical classes (hydrocarbons and ester groups) were absent in this sample. The compositions of the essential oils isolated from* R. chalepensis* from the three regions of Palestine are illustrated in Table 1.

### 3.1. Antioxidant Property

**Figure 1:**
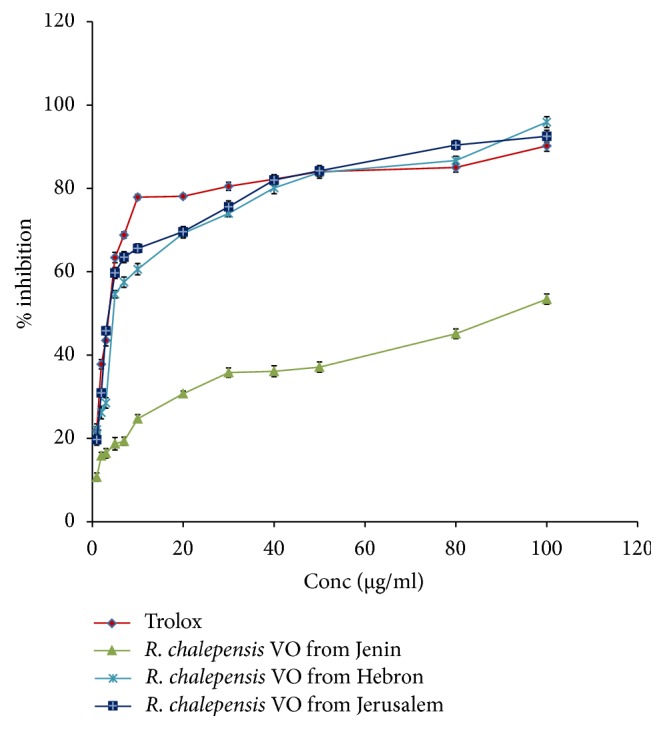
The antioxidant activity of Trolox standard and the three studied* R. chalepensis* essential oils.

The antioxidant activity of the essential oil of* R. chalepensis*, collected from three regions of the Palestinian West Bank, was tested by DPPH and using Trolox as a reference compound. The used concentrations ranged 1–100 *μ*g/ml for all the studied essential oils from three regions as well as for standard Trolox as shown in Figure 1. The results revealed that the free radical scavenging property was exhibited by all the studied* R. chalepensis* essential oils. IC_50_ and percentages of inhibition for* R. chalepensis* collected from Jerusalem, Hebron, and Jenin were 6.9 ± 0.94 *μ*g/ml, 69.56%, 7.8 ± 1.05 *μ*g/ml, 61.53%, and 19.9 ± 0.68 *μ*g/ml 24.12%, respectively.

The calculated IC_50_ and percentages of inhibition for* R. chalepensis* essential oils from three regions according to Trolox standard antioxidant molecule represented in Table 2.

### 3.2. Antimicrobial Activity

The essential oil of* R. chalepensis* from Jerusalem, Hebron, and Jenin regions of Palestine extracted by microwave-ultrasonic method exhibited interesting potential bioactivity against the growth of all microbes examined in this study. However, the highest antibacterial activity (lowest MIC) against bacterial pathogen examined was for* R. chalepensis* essential oil from Jerusalem and was seen against* E. coli*,* P. aeruginosa*,* S. aureus*, and MRSA with MIC values of 0.75 mg/ml, 7 mg/ml, 2.5 mg/ml, and 4 mg/ml, respectively, in comparison with gentamycin which has MIC values of 0.5 mg/ml, 2 mg/ml, 0.5 mg/ml, and 1.5 mg/ml, respectively. On the other hand, the best antifungal activity against* C. albicans* was for* R. chalepensis* essential oil from Jenin region which showed MIC value close to 2.75 mg/ml in comparison with amphotericin B which had MIC value around 2 mg/ml. The antifungal and antibacterial activity for the three essential oils are shown in Table 3.

## 4. Discussion

Palestine has an unique geographical location which results in an unique biodiversity as well. In fact, it is located at the meeting point of three continents: Europe, Asia, and Africa. It extends between the Mediterranean Sea and the continental rift valley which includes the Dead Sea, the most unique sea in the world since it is located in the lowest level from the sea. In addition, it has a lot of mountains, plains, and a desert which also results in an unique weather and climate. Accordingly, the Palestinian desert, plains, and mountains are rich in plants of various species. In fact, more than 2700 plants species are distributed on this small Mediterranean area, of which more than 300 are mentioned in published ethnobotanical data and its cultural characteristics are highlighted in the literature, including traditional herbal Palestinian medicine and materia medica for curing various illnesses [47]. As reported by Salgueiro et al., in 1997 [48], climate, genotype, growth area, rainfall, and harvest methods can influence the essential oil composition of plants. Many published studies demonstrated that the yield and chemical composition of the essential oils varied within different geographical areas [24, 30, 49–52].

The essential oils obtained from* R. chalepensis* growing in different countries have shown very large differences in their major components. The 2-undecanone and 2-nonanone are the major components of the essential oil from most of the studied* R. chalepensis* leaves from various countries [53–57].

A study which was performed by Tzakou and Couladis, 2011, on the essential oil of* R. chalepensis* from Greece showed that the main components of the oil were *β*-phellandrene (10.7%) and 2-methyloctyl acetate (44.0%) [58]. Another study which was conducted in Tunisia by Tounsi et al., 2011, showed that the leaves of cultivated* R. chalepensis* contained 2-nonanol as the major active constituents of the essential oil [59]. Another study conducted also in Tunisia by Ghazghazi et al., 2015, showed that the main chemical constituents of* R. chalepensis* essential oil were menthol (43.92%) and linalool (42.10%) [60]. These variations in the phytochemical components may be due to the various geographical locations and climatic and weather conditions from which the* R. chalepensis* leaves were collected.

In fact, a high similarity was observed in the quality and the biological activities of the essential oils obtained from the Hebron and Jerusalem mountains, while the essential oil obtained from Jenin region showed different quality and activity. This may be due to differences in the location and weather between these regions. In fact, Jenin is a plain area and it is located in the northern part of Palestine. In addition, it has a very humid and hot weather. On the other hand, both Hebron and Jerusalem are mountainous regions and are located in the middle to southern area of the West Bank, close to the Dead Sea, and they usually have moderate to dry weather. Moreover, the soil in Palestine is highly variable in type and characteristics. Brown rendzinas and pale rendzinas are found in the southern mountain ridge, in Jerusalem and Hebron, whereas grumosols are found Jenin within the northern West Bank, which is known for low-lying areas that exhibit a higher temperate climate compared to other regions [61]. These variations in soil type and climate may affect the growth and characteristics of the native flora.

More specifically, the antioxidant activity results showed that the essential oil obtained from* R. chalepensis* leaves which were collected from Jerusalem region had the highest antioxidant potential followed by the essential oil obtained from Hebron regions with antioxidant potential of 69.56% and 61.53% of inhibition according to Trolox; meanwhile the antioxidant potential was the weakest for the essential oil obtained from* R. chalepensis* from Jenin region which had 24.12% of inhibition potential compared to Trolox a reference antioxidant compound.

To the best of our knowledge, only one research was performed to assess the antioxidant activity of* R. chalepensis.* In this study, the method of extraction was Soxhlet with methanol/water, and it was not conducted on the essential oil nor using microwave sonicator as method of extraction. The reported antioxidant activity was several times lower than our results (IC_50_ = 70.01 *μ*g/ml) [62].

In fact, our tested* R. chalepensis* essential oil from Jerusalem, Hebron, and Jenin regions had IC_50_ = 6.9 ± 0.94 *μ*g/ml, 7.8 ± 1.05 *μ*g/ml, and 19.9 ± 0.68 *μ*g/ml, respectively, that showed that our studied essential oils had more powerful antioxidant activity than the above-mentioned study on the antioxidant potential for* R. chalepensis* leaves extract [62]. This may be justified due to differences in the location, weather, soils, method of preparation, and type of tested extracts.

Regarding the antiseptic activities, the best antibacterial activity of* R. chalepensis* essential oil against* E. coli*,* P. aeruginosa*,* S. aureus*, and MRSA was for the essential oil obtained from the plant from the Jerusalem region followed by the essential oil obtained from Hebron region which had MIC values of 1.5 mg/ml, 9 mg/ml, 3.25 mg/ml, and 8 mg/ml, respectively.

In addition, the weakest antibacterial activity was for the essential oil obtained from Jenin region with MIC values of 12 mg/ml, 22 mg/ml, 10 mg/ml, and 21 mg/ml, respectively.

Regarding the antifungal activity for* R. chalepensis* leaves essential oil was the best for the plants collected from Jenin region and was 2.75 mg/ml followed by the essential oil from Jerusalem region while the lowest antifungal potential was for the essential oil obtained from Hebron.

These results about antifungal activity had been demonstrated in a study conducted by Khoury et al., 2014, which showed that the essential oil from* R. chalepensis* growing in Lebanon had the major components 2-nonanone, 51.7%, and 2-undecanone, 36.7%, which exhibited antifungal activity against the yeast* Candida albicans* and the dermatophyte* Trichophyton rubrum* [54].

All the antioxidant and antibacterial results may be explained by the fact that the essential oils from Jerusalem and Hebron are rich in linalyl acetate which showed powerful antibacterial activity and can result in alterations of membrane permeability and in leakage of intracellular substances of microorganisms. In addition, linalyl acetate might interact with intracellular sites resulting in antibacterial activity [41, 63].

The essential oil of* R. chalepensis* plant from Jerusalem and Hebron had high content of linalyl acetate which corresponded with the best antibacterial activity while the essential oil from Jenin area did not contain this pharmacologically active compound. For that reason, this plant essential oil had the lowest antibacterial activity.

Further pharmacological and clinical studies are required to evaluate the effects of* R. chalepensis* essential oil collected from Jerusalem and Hebron regions against cancer, Alzheimer's disease, and cardiovascular and other diseases. In elaboration of the above, the essential oil of* R. chalepensis* from Jenin region also needs more studies on other fungal infections to evaluate the complete antifungal activity of this plant species.

## 5. Conclusion

The results showed that* R. chalepensis* collected from the mountains of Jerusalem and Hebron regions of Palestine had almost the same essential oil constituents and had powerful antioxidant and antibacterial activities; meanwhile the plant species growing wildly in Jenin had different essential oil constituents and had lower antioxidant and antibacterial activities but had potential antifungal activity. Furthermore, our results indicated that the geographical location and ecological conditions affected the chemical constituents of the essential oil. Our investigation could have numerous applications in nutraceutical, pharmaceutical, and food and cosmetic industries. Finally the phytoconstituents and antioxidant and antibacterial properties of the essential oil of wild* R. chalepensis* growing in Palestine are now well established in this study which could indicate the future uses of* R. chalepensis* in the treatment of various infectious and noninfectious diseases in addition to other ailments. In addition, this study highlighted the importance of fixing all variables that might affect the final composition of the extract including area of cultivation, climatic and weather conditions, method of preparation, and the solvent used for extraction.

## Figures and Tables

**Table 1:** Chemical composition, concentrations (%), and calculated retention indices of *R. chalepensis* essential oils as characterized by GC-MS analysis.

Compound	% of total essential oil from Hebron	% of total essential oil from Jerusalem	% of total essential oil from Jenin	The mean of calculated retention Index (Kovats)
Linalyl acetate	29.51	34.21	—	1259
*β*-Linalool	26.79	31.78	—	1103
2-Undecanone	14.99	7.66	44.31	1297
2-Nonanone	14.29	8.15	43.02	1093
*α*-Terpineol	3.78	4.17	—	1197
Camphor	1.58	1.82	0.47	1149
Isocaryophyllene	1.58	1.72	—	1404
1,8-Cineole	1.52	1.84	0.84	1033
L-4-Terpineol	0.7	0.96	—	1184
Limonene	0.62	1.18	—	1030
Isoborneol	0.57	0.66	—	1172
3-Octanone	0.3	0.64	—	988
1-Octen-3-ol	0.27	0.39	—	982
1-Heptanol	0.25	0.14	0.53	973
n-Hexyl acetate	—	0.66	—	1013
Alpha cis-Ocimene	—	0.18	—	1039
3-Octanol	—	0.14	—	997
2-Nonanol	—	—	1.4	1102
2-Decanone	—	—	2.01	1196

Total identified components (%)	96.75	96.3	92.58	

*Grouped components*				
Hydrocarbons	2.2	2.9		
Alcohols	32.36	38.24	1.93	
Ketones	31.16	18.27	89.81	
Esters	29.51	35.05		
Oxide	1.52	1.84	0.84	

Total identified chemical classes (%)	96.75	96.3	92.58	

**Table 2:** The antioxidant activity (IC_50_) and percentage of inhibition for* R. chalepensis* essential oils from three regions according to the Trolox a reference compound.

Methanolic extract	Log IC_50_ (*μ*g/ml) (Mean ± SD)	Inhibition (%) according to the Trolox
*R. chalepensis *essential oil from Jenin	19.9 ± 0.68^*∗∗*^	24.12
*R. chalepensis *essential oil from Hebron	7.8 ± 1.05^*∗*^	61.53
*R. chalepensis *essential oil from Jerusalem	6.9 ± 0.94^*∗∗∗*^	69.56
Trolox	4.8 ± 0.51^*∗*^	100

^*∗*^ *p* value < 0.05, ^*∗∗*^*p* value ≤ 0.001, and ^*∗∗∗*^*p* value ≤ 0.0001.

**Table 3:** Antimicrobial activities for *R. chalepensis *three studied essential oils.

Studied samples	*E. coli* (ATCC 25922)	*P. aeruginosa* (ATCC 27853)	*S. aureus *(ATCC 25923)	MRSA	*C. albicans*
*R. chalepensis *essential oil from Jenin	12	22	10	21	2.75
*R. chalepensis *essential oil from Jerusalem	0.75	7	2.5	4	3.5
*R. chalepensis *essential oilfrom Hebron	1.5	9	3.25	8	4
Gentamycin (antibacterial reference)	0.5	2	0.5	1.5	—
Amphotericin B (antifungal reference)	—	—	—	—	2

## Abbreviations

GC-MS: Gas chromatography mass spectrometry
ATCC: American Type Culture Collection
DPPH: 2,2-Diphenyl-1-picrylhydrazyl
DMSO: Dimethyl sulfoxide
MIC: Minimal inhibitory concentration
IC_50_: Half-maximal inhibitory concentration.
